# Astrocytes: GABAceptive and GABAergic Cells in the Brain

**DOI:** 10.3389/fncel.2022.892497

**Published:** 2022-06-10

**Authors:** Jianhui Liu, Xuanran Feng, Yi Wang, Xiaohuan Xia, Jialin C. Zheng

**Affiliations:** ^1^Department of Anesthesiology, Tongji Hospital affiliated to Tongji University School of Medicine, Shanghai, China; ^2^Translational Research Center, Shanghai Yangzhi Rehabilitation Hospital affiliated to Tongji University School of Medicine, Shanghai, China; ^3^Center for Translational Neurodegeneration and Regenerative Therapy, Tongji Hospital affiliated to Tongji University School of Medicine, Shanghai, China; ^4^Shanghai Frontiers Science Center of Nanocatalytic Medicine, Shanghai, China; ^5^Translational Research Institute of Brain and Brain-Like Intelligence, Shanghai Fourth People’s Hospital affiliated to Tongji University School of Medicine, Shanghai, China; ^6^Collaborative Innovation Center for Brain Science, Tongji University, Shanghai, China

**Keywords:** astrocyte, GABA, brain, neuron, microglia, gliotransmitter

## Abstract

Astrocytes, the most numerous glial cells in the brain, play an important role in preserving normal neural functions and mediating the pathogenesis of neurological disorders. Recent studies have shown that astrocytes are GABAceptive and GABAergic astrocytes express GABA_A_ receptors, GABA_B_ receptors, and GABA transporter proteins to capture and internalize GABA. GABAceptive astrocytes thus influence both inhibitory and excitatory neurotransmission by controlling the levels of extracellular GABA. Furthermore, astrocytes synthesize and release GABA to directly regulate brain functions. In this review, we highlight recent research progresses that support astrocytes as GABAceptive and GABAergic cells. We also summarize the roles of GABAceptive and GABAergic astrocytes that serve as an inhibitory node in the intercellular communication in the brain. Besides, we discuss future directions for further expanding our knowledge on the GABAceptive and GABAergic astrocyte signaling.

## Introduction

Astrocytes, the most abundant glial cells in the central nervous system (CNS), account for about 20% of the glial cells in the neocortex of the human brain (Pelvig et al., 2008). They form complex connections with neurons, blood vessels, and other glial cells, and play an important role in preserving normal brain functions *via* providing energy and nutritional support for neurons, maintaining metabolic homeostasis of the CNS, and regulating cerebral blood flow (Anderson and Nedergaard, 2003; Bélanger et al., 2011; Scheiber and Dringen, 2013).

A single astrocyte can touch more than 100,000 synapses in the mouse cortex *via* a tripartite synapse, a structure that the astrocyte associates with the pre- and post-synapse areas of neurons (Bushong et al., 2002). Astrocytes induce synaptic formation, regulate the release and uptake of synaptic neurotransmitters, and maintain synaptic cleft transmitter homeostasis (Allen, 2014). More importantly, astrocytes directly regulate synaptic plasticity and synaptic transmission by releasing gliotransmitters such as glutamate, adenosine triphosphate (ATP), taurine, glycine, and D-serine (Barakat and Bordey, 2002; Hussy, 2002; Henneberger et al., 2010; Bernardinelli et al., 2014; Mederos and Perea, 2019). In the recent decade, gamma-aminobutyric acid (GABA) has emerged as a novel gliotransmitter (Yoon and Lee, 2014). Astrocytes express GABA receptors to interact with an extracellular GABA, suggesting astrocytes as GABAceptive cells (Le Meur et al., 2012; Yoon et al., 2012). Astrocytes also contain a considerable amount of GABA that can be released to modulate the activities of GABA receptors-expressing cells, indicating a GABAergic role of astrocytes (Le Meur et al., 2012; Yoon et al., 2012).

## Astrocytes Are GABAceptive Cells

### Astrocytes Internalize GABA *via* GABA Receptors and Transporters

**Figure 1:**
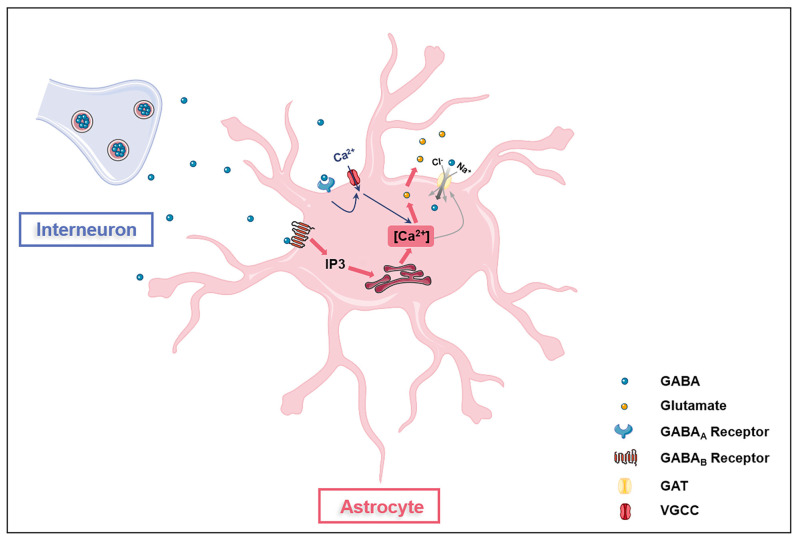
Astrocytes are GABAceptive cells. Astrocytes express GABA_A_ receptors (GABA_A_Rs), GABA_B_ receptors (GABA_B_Rs), and GABA transporter proteins (GATs). Activation of GAGA_A_Rs on astrocytes opens voltage-gated calcium channels (VGCC), leading to the influx of extracellular Ca^2+^ into the cell (blue arrow). GAGA_B_Rs activation induces the release of Ca^2+^ from the intracellular IP3-sensitive Ca^2+^ pool (red arrow). Ca^2+^ oscillations in astrocytes affect glutamate release and GATs expression. GATs directly transport extracellular GABA into astrocytes.

GABA is the main inhibitory transmitter in adults, which binds to GABA receptors (ionotropic GABA_A_ receptors, GABA_A_Rs and metabotropic GABA_B_ receptors, GABA_B_Rs) on neurons and inhibits neuronal activities *via* reducing exocytosis, hyperpolarizing membranes, and shunting depolarization. Astrocytes are also with GABA uptake capacity which requires at least two sodium ions per transportable GABA molecule (Figure 1; Larsson et al., 1980). Astrocytes express both GABA_A_Rs and GABA_B_Rs in the soma, the synapse-surrounding processes, and the brain vessel-contacting endfeet (Nilsson et al., 1993; Charles et al., 2003; Meier et al., 2008). The GABA_A_Rs consist of five protein subunits arranged around a central pore that constitutes the ion channel. Each subunit has a large extracellular N-terminal domain, three membrane spanning domains (M1-3), an intracellular loop of variable length, and a fourth membrane spanning domain (M4) with extracellular C-terminal end. The GABA_A_R family comprises 19 discovered subunits: α_1–6_, β_1–3_, γ_1–3_, ρ_1–3_, δ, ε, π, and θ, and the subunit combinations lead to a great diversity of GABA_A_Rs (Olsen and Sieghart, 2008, 2009; Sequeira et al., 2019). Indeed, about 20 widely occurring native GABA_A_Rs have been identified, with the major combinations being α_1_β_2/3_γ_2_, α_2_β_3_γ_2_, α_3_β_3_γ_2_ (Barnard et al., 1998; Mohler, 2006). The structure diversity confers GABA_A_Rs with distinct topology, channel kinetics, affinity for GABA, rate of desensitization, and ability for transient chemical modification such as phosphorylation (Mohler, 2006). Although the exact types of astroglial GABA_A_Rs in the brain have not been clearly distinguished, functional GABA_A_Rs have been found on astrocytes (Fraser et al., 1995). The mRNAs of many GABA_A_Rs subunits including α_1–5_, β_1–3_, γ_1–3_, and δ have been detected in cultured primary astrocytes isolated from rodent cerebella (Bovolin et al., 1992; Zheng et al., 1993). A recent study on human brains reported expressions of genes encoding α_2_, β_1_, and γ_1_ subunits in astrocytes, indicating the existence of functional astroglial α2β1γ1 receptors in humans (Sequeira et al., 2019). Extracellular GABA can activate astroglial GABA_A_Rs to open Cl^−^ channels in astrocytes in primary cell culture and rodent hippocampal slices (Kettenmann et al., 1987; MacVicar et al., 1989). The Cl^−^-mediated depolarization results in an influx of Ca^2+^ from the extracellular space through L- and T-type voltage-gated calcium channels (VGCC; Young et al., 2010). GABA also activates astroglial GABA_B_Rs. Unlike GABA_A_Rs, GABA_B_Rs, belonging to class C of G-protein coupled receptors (GPCRs), mediate slow and prolonged inhibitory signaling in the brain *via* the activation of Gi/o type G-proteins, thus lead to inhibition of adenylyl cyclase (AC; Munk et al., 2016). GABA_B_Rs are obligate heterodimers composed of GABA_B1_ and GABA_B2_ subunits (Evenseth et al., 2020). Each subunit consists of an extracellular Venus flytrap (VFT) domain and a heptahelical transmembrane (7TM) domain (Chun et al., 2012). The VFT is connected to the 7TM by a linker without the cysteine rich domain (CRD; Chun et al., 2012). There are multiple isoforms of the GABA_B1_ subunit, but the most abundant are GABA_B1a_ and GABA_B1b_, encoded by the same gene, *GABBR1* (Kaupmann et al., 1997). GABA_B1a_, GABA_B1b_, and GABA_B2_ receptor subunits are all expressed on astrocytes (Charles et al., 2003). The activation of astroglial GABA_B_Rs increases intracellular Ca^2+^, which triggers the release of Ca^2+^ from intracellular IP_3_-sensitive Ca^2+^ pools (Lee et al., 2011; Vélez-Fort et al., 2012; Mariotti et al., 2016). Gamma-hydroxybutyric acid, a metabolite of GABA, also activates GABA_B_Rs, which in turn stimulates astrocytes (Gould et al., 2014). GABA is depolarizing in astrocytes as their chloride equilibrium potential is more depolarized than their resting membrane potential due to the lack of chloride-extruding transporter, potassium chloride cotransporter 2 (KCC2; Kolta, 2018). The expression of GABA receptors in astrocytes is affected by many factors. For instance, GABA_A_Rs in astrocytes decrease with *in vitro* aging and cerebral ischemia, possibly due to the overproduction of S100B in activated astrocytes (Tateishi et al., 2006).

Besides, astrocytes express multiple GABA transporter proteins (GATs), including GAT-1, GAT-3, and betaine-GABA transporter (BGT-1; Schousboe et al., 2017). Although GAT-1 is predominantly expressed in GABAergic neurons for the recycling of GABA in presynapse (Conti et al., 2004), the GAT-1 subtype is found in astrocytes to be responsible for glial GABA transport (Radian et al., 1990; Schousboe et al., 2017). Unlike GAT-1, GAT-3 is expressed exclusively by astrocytes and is mainly located at the astroglial processes to modulate tonic inhibitory currents in postsynaptic cells (Durkin et al., 1995; Minelli et al., 1996; Kersante et al., 2013; Melone et al., 2015). GAT-3 activities influence various astroglial functions including astrocyte synaptic proximity, inhibitory synapse efficacy regulation, excitatory neurotransmission modulation, and heroin seeking, indicating GAT-3 as a key glial GABA transport (Shigetomi et al., 2011; Boddum et al., 2016; Kruyer et al., 2021). Studies have reported conflicting results regarding the expression of BGT-1 in astrocytes (Bitoun and Tappaz, 2000; Olsen et al., 2005; Zhou et al., 2012; Schousboe et al., 2017). BGT-1 expression can be detected in cultured astrocytes (Olsen et al., 2005), however, the expression of BGT-1 in astrocytes *in vivo* may be very low (Bitoun and Tappaz, 2000; Zhou et al., 2012; Schousboe et al., 2017). Thus, GAT-1 and GAT-3 appear to be the two GABA transporters that are mainly responsible for GABA uptake (Kersante et al., 2013) and intracellular Ca^2+^ signaling of astrocytes (Doengi et al., 2009; Matos et al., 2018), even though BGT-1 may also play a role (Schousboe et al., 2017). Interestingly, the levels of Ca^2+^ signaling influence GATs expression as the enhanced extrusion of cytosolic Ca^2+^ *via* plasma membrane Ca^2+^ pump PMCA2 upregulates GATs expression in astrocytes (Yu et al., 2018). Hence, aforementioned literatures indicate that astroglial GATs act in concert with GABA receptors to regulate extracellular GABA levels in the brain.

### GABA Regulates the Differentiation, Metabolism, and Functions of Astrocytes

GABA plays an important role in the differentiation, maturation, and morphology of astrocytes. GABA treatment significantly promotes the morphological differentiation of neonatal and adult astrocytes *in vitro* and *in vivo* (Matsutani and Yamamoto, 1997; Mong et al., 2002; Runquist and Alonso, 2003). The effects can be blocked by GABA_A_ antagonist, suggesting the involvement of GABA_A_R in GABA-induced neonatal astrocyte differentiation (Matsutani and Yamamoto, 1997).

GABA also influences the metabolism of astrocytes. GABA released by starvation- or ghrelin treatment-activated agouti-related protein (AgRP) neurons can replace glutamate as an energy source and affect the metabolic fate of glutamate and glucose in astrocytes, thus inducing the depolarization of astrocytes, expression of the glial fibrillary acidic protein (GFAP), and mitochondrial fission (McKenna and Sonnewald, 2005; Varela et al., 2021). GABA metabolism in astrocytes is perturbed in neurological disorders. GABA metabolism has been found to be downregulated in astrocytes with APP or PSEN-1 mutations, which was associated with the decline of GAT-3 expression and GABA uptake (Salcedo et al., 2021). In cortical astrocytes, both the nitrogen and carbon skeleton of GABA can be used for glutamine synthesis (Andersen et al., 2020). Although exogenous GABA may not directly stimulate glycolysis or oxidative metabolism in astrocytes, it is used as an additional substrate for uncoupled respiration to enhance this reaction.

The functions of astrocytes are under the regulation of GABA as well. Astrocytes are an important unit of blood-brain-barrier (BBB) *via* the interaction of endothelial cells (Abbott et al., 2006). GABA released by interneurons in the basal forebrain (BF) activates GABA_A_Rs in astrocytes that attach to microvessels or vascular walls, thus inducing astrocyte-mediated vascular dilation (Kaupmann et al., 1997). Similarly, astrocyte GABA uptake trigger vasoconstriction in developing olfactory bulb (Vélez-Fort et al., 2012).

Except for BBB regulation, GABA also influences the molecule release capacity of astrocytes. GABA inhibited cultured rat astrocytes from releasing endozepine (Patte et al., 1999). Endozepines are a family of astroglia-secreted proteins, namely diazepam-binding inhibitor/acyl-CoA-binding protein (DBI/ACBP) and its processing fragments, triakontatetraneuropeptide (TTN), and octadecaneuropeptide (ODN; Guidotti et al., 1983; Knudsen et al., 1989; Rothstein et al., 1992; Farzampour et al., 2015; Masmoudi-Kouki et al., 2018; Lebrun et al., 2021). Although the term endozepines has been around 40 years, endozepines remain a controversial theme due to unclarity of the exact roles of proposed endozepines in the brain (Tonon et al., 2020). Endozepines have been originally isolated and characterized as natural ligands of central-type benzodiazepine receptor (BZR), located on the GABA_A_ receptor complex (Guidotti et al., 1983). Following studies have reported that endozepines interact with another BZR, the mitochondrial translocator protein (TSPO; Slobodyansky et al., 1989), and GPCR coupled to the PLC/PKC and/or AC/PKA pathways (Patte et al., 1995; Marino et al., 2003). Afterwards, growing evidence has strongly suggested that endozepines act as endogenous regulators of anxiety-related behaviors (Guidotti et al., 1983; De Mateos-Verchere et al., 1998), energy balance (Guillebaud et al., 2017; Lebrun et al., 2021), neuroprotection (Ghouili et al., 2018; Masmoudi-Kouki et al., 2018), neurogenesis, tumorigenesis (Dumitru et al., 2017; Duman et al., 2019), and hormonal secretions (Yoshida et al., 1999; Tonon et al., 2020). It is also worth-noting that the identification of endozepines is still an ongoing work. There are other endozepines such as endozepine-2 and endozepine-4 that may be associated with the pathogenesis of stupor (Rothstein et al., 1992). Hence, GABA modulates astroglial function under physiological and pathological conditions *via* inducing endozepines secretion.

Besides, GABA also regulates the release of ATP and adenosine from astrocytes, which, modulates neuronal function (Orellana and Stehberg, 2014; Matos et al., 2018). Astroglial ATP acts on presynaptic P2X receptors to trigger a prolonged increase of GABA release, therefore switching the plasticity of GABA synapses in the dorsomedial hypothalamus (Crosby et al., 2018). The GABA-driven release of astroglial adenosine acts on presynaptic A1 receptors to mediate heterosynaptic depression and propagation of glial activation (Newman, 2003; Serrano et al., 2006), therefore regulating mnemonic processes (Vogt and Nicoll, 1999; Guetg et al., 2009) and the pathogenesis of various neurological disorders including epilepsy (Maitre et al., 1974; Heja, 2014).

### GABAceptive Astrocytes Fine-Tune Astrocyte-Neuron Crosstalk

**Figure 2:**
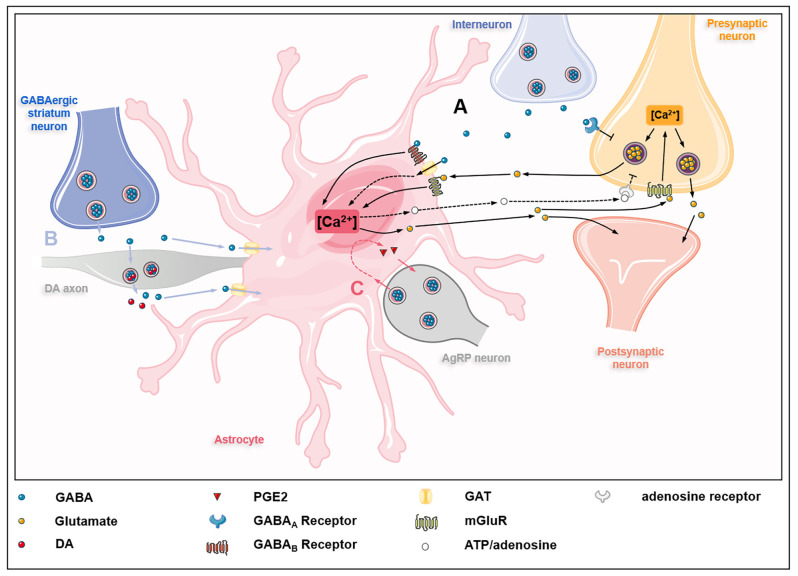
GABAceptive astrocytes fine-tune astrocyte-neuron crosstalk. **(A)** GABA released by interneurons on one hand inhibits glutamate release from presynaptic neurons by acting on GABA_A_ receptors (GABA_A_Rs) on presynaptic neurons; on the other hand, GABA acts on GABA_B_ receptors (GABA_B_Rs), metabotropic glutamate receptors (mGluRs), and GABA transporter proteins (GATs) on astrocytes, causing Ca^2+^ oscillations in astrocytes. Ca^2+^ oscillations regulate the release of glutamate and ATP/adenosine. Glutamate derived from astrocytes acts on mGluRs on presynaptic neurons, thus promoting the release of glutamate from presynaptic neurons into the synaptic cleft. In addition, astrocyte-derived glutamate also acts directly on postsynaptic neurons to partially counteract the inhibitory effect of GABA. Besides, astrocyte-derived ATP/adenosine inhibits glutamate release from presynaptic neurons by activating presynaptic adenosine receptors (dashed lines). **(B)** GABA released by GABAergic striatum neurons acts on dopamine (DA) axons and inhibits the co-release of GABA and dopamine, which is modulated by GATs on astrocytes (blue lines). **(C)** GABA released by agouti-related protein (AgRP) neurons acts on astrocytes, causing astrocytes to release prostaglandin E_2_ (PGE2) to activate AgRP neurons (red lines).

The GABA uptake capacity confers astrocytes an essential role in regulating inhibitory networks in the brain (Figure 2). Interneuron-derived GABA increases GAT-1 and GAT-3 activity in astrocytes, which modulates synaptic activities of thalamocortical neurons and striatal output neurons, thus maintaining the tonic inhibition in the thalamus and striatum, respectively (Pirttimaki et al., 2013; Wójtowicz et al., 2013; Boddum et al., 2016). The activities of GAT-1 and GAT-3 also influence GABA_A_R-mediated inhibitory transmission (Moldavan et al., 2017). The inhibition or knockout of GATs induces the accumulation of extracellular GABA in the brain, leading to extrasynaptic GABA_A_Rs activation and GABA_A_R-mediated tonic current induction (Chiu et al., 2005; Song et al., 2013). Besides, astrocytes from the somatosensory cortex and the hippocampus can sense GABA released from parvalbumin (PV)-expressing interneurons *via* GABA_B_R, which influences inhibitory post-synaptic current potentiation at the medial prefrontal cortex (mPFC) circuits (Perea et al., 2016; Covelo and Araque, 2018; Mariotti et al., 2018; Mederos et al., 2021). The GABA_B_R-mediated coordination of excitation-inhibitory balance and gamma oscillations plays an important role in goal-directed behaviors (Mederos et al., 2021). The response of astrocytes to GABA signals depends on the type of interneurons involved. The synaptic activities of somatostatin-expressing interneurons (SOM-INs) can be detected by hippocampal astrocytes *via* GABA_B_R and GAT-3-dependent Ca^2+^ signaling mechanisms, leading to the release of ATP and the production of adenosine to activate SOM-IN synaptic inhibition of pyramidal cells (Mariotti et al., 2016; Matos et al., 2018; Losi et al., 2021). GABA-stimulated astrocytes also release prostaglandin E_2_ (PGE2) to activate AgRP-expressing neurons *via* EP2 receptor (Varela et al., 2021). Astrocytes also regulate dopaminergic neurotransmission by controlling extracellular uptake of GABA (Roberts et al., 2020). Under normal circumstances, GABA released by GABAergic striatum neurons acts on GABA receptors located in dopamine (DA) axons, thereby inhibiting the co-release of DA and GABA in DA axons. This process is controlled by the activity of astrocyte GATs which internalize GABA from extracellular space.

Interestingly, the activation of GABAceptive astrocytes has effects on excitatory neurotransmission as well. Astrocyte GAT3-mediated regulation of extracellular GABA in the hippocampus plays an important role in controlling the excitability of hippocampal cells in response to increased network activity (Kersante et al., 2013). The GABA-induced activation of GABA_B_Rs results in glutamate release from astrocytes and activation of presynaptic group I metabotropic glutamate receptors (mGluRs), which persists in the bursts of interneuron action potential seven during the interneuron down-state, leading to enhancement of excitatory neurotransmission (Perea et al., 2016). Furthermore, computational modeling has recently been utilized to investigate the effects of exposure of astrocytes to different concentrations of exogenous GABA on excitatory presynaptic and postsynaptic endings (Li et al., 2020). The results show that increased GABA concentration not only reduces neuronal spikes but also facilitates astrocyte glutamate release by inducing Ca^2+^ oscillations, leading to astrocyte-mediated presynaptic release and enhanced postsynaptic slow inward current (i.e., depolarizing currents). Thus GABA-activated astrocytes induce neuronal excitation that partially counteracts GABA inhibition.

In addition, GABA-stimulated astrocytes can affect the differentiation and maturation of neurons. During early development, astrocytes internalize neuronal precursor-derived GABA to create a microenvironment that strictly regulates the level of GABA and the activation of GABA_A_R, which is conducive to controlling the migration rate of neuronal precursors during development (Bolteus and Bordey, 2004).

### GABA-Stimulated Astrocytes Contribute to Neurological Disorders

GABA neurotransmission disorders have been reported in various neurological diseases. For instance, the decline of GABA uptake and metabolism in AD astrocytes leads to GATs expression reduction, hereby contributing to neurotransmitter disturbances and cognitive impairment (Salcedo et al., 2021). In mouse models of early Parkinson’s disease, the downregulated GATs expression in the dorsal striatum also reduces the co-release of DA and GABA in DA axons, enhancing tonic inhibition of DA release and accelerating disease progression (Roberts et al., 2020). Furthermore, the activation of astrocyte GABA_B_R by striatal medium spiny neurons results in acute behavioral hyperactivity and disrupted attention, revealing the activation of GABAceptive astrocytes as a causal factor for hyperactivity, attention deficit, and related psychiatric disorders (Nagai et al., 2019). In hyperammonemia, astrocyte activation and neuroinflammation have been reported to participate in GABA neurotransmission alteration, which causes cognitive dysfunction in hepatic encephalopathy (Malaguarnera et al., 2019, 2021). Treatment by GABA_A_R antagonist bicuculline can restore GABA neurotransmission, leading to the recovery of spatial learning and reduction of anxiety (Malaguarnera et al., 2019, 2021). Moreover, enhanced GABA_A_R responses of astrocytes are required for endozepines actions in the thalamic reticular nucleus (nRT), which mediates antiepileptic and sleep-promoting effects (Christian and Huguenard, 2013). Besides, dysfunction of GABAceptive astrocytes alters GABAergic transmission, thus contributing to epilepsy.

Taken together, astrocytes express GABA receptors and transporters. The uptake of GABA regulates astrocyte differentiation and function, therefore influencing GABA neurotransmission and contributing to neurological disorders.

## Astrocytes Are GABAergic Cells

### Astrocytes Produce and Release GABA

**Figure 3:**
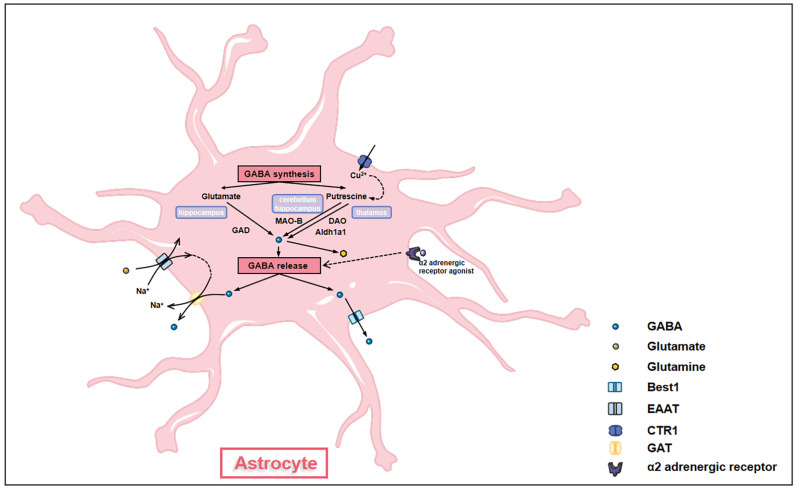
Astrocytes synthesize and release GABA. GABA synthesis in astrocytes has different pathways in different brain regions. In the hippocampus, glutamate in astrocytes is converted into GABA under the action of glutamate acid decarboxylase (GAD). In the cerebellum and the hippocampus, putrescine in astrocytes is converted into GABA under the action of monoamine oxidase B (MAO-B). In the thalamus, putrescine in astrocytes generates GABA through a two-step interaction of diamine oxidase (DAO) and aldehyde dehydrogenase 1 family A1 (Aldh1a1). The transformation of putrescine to GABA is affected by the levels of intracellular copper ion which is regulated by copper transporter (CTR). GABA synthesized by astrocytes can be further converted into glutamine to enter glutamine-glutamate cycle. GABA is released out of cells by either GABA transporter proteins (GATs) under the action of glutamate transporter (EAAT) or through bestrophin (Best1). α-2 adrenergic receptors also modulate the release of GABA through G_iβγ_ subunit-associated signaling pathways.

GABA was previously thought to be produced in and released from neurons only. However, emerging evidence has demonstrated that similar to neurons, astrocytes are capable of producing and releasing GABA, suggesting astrocytes as GABAergic cells (Figure 3; Le Meur et al., 2012). Astrocytes synthesize GABA using diverse enzymes like monoamine oxidase B (MAO-B) and glutamic acid decarboxylase (GAD) in different brain regions (Wu et al., 2014; Yoon et al., 2014). In the thalamus, diamine oxidase (DAO) and aldehyde dehydrogenase 1 family A1 (Aldh1a1) in astrocytes convert putrescine to GABA through two metabolic steps and release by bestrophin (Kwak et al., 2020). In the cerebellum and hippocampus, MAO-B converts putrescine into GABA in astrocytes (Yoon et al., 2014; Park et al., 2019). Besides, hippocampal astrocytes utilize two glutamate decarboxylases (GAD65 and GAD67, 65- and 67-kD isoforms, respectively) to convert glutamate into GABA (Kwak et al., 2020). Putrescine in astrocytes can be catabolized to GABA under the catalysis of copper amine oxidases (CAOs; Szabó et al., 2021).

Astrocytes release GABA post various stimulations. For example, intracellular copper levels affect the amount of GABA accumulated in astrocytes. After being taken up by copper transporter (CTR1), copper enhances CAOs’ activities and GABA production, causing GABA release and tonic inhibition (Szabó et al., 2021). Astrocyte GABA can also be released into the extrasynaptic space by Na^+^ influx mediated by glutamate transporter (EAAT; Szabó et al., 2021). Besides, activation of α-2 adrenergic receptors also stimulates the release of GABA by astrocytes through G_iβγ_ subunit-associated signaling pathways (Gaidin et al., 2020a). Thus, the GABAceptive and GABAergic characteristics confer essential roles of GABA homeostasis maintenance to astrocytes, making GABAergic astrocytes key nodes of inhibitory networks in the brain. Moreover, astrocyte GABA is co-released with glutamate, acetylcholine, dopamine, or histamine in the presynaptic terminal, providing temporal-spatial precise signals and regulating synaptic plasticity (Tritsch et al., 2016).

### GABAergic Astrocytes Contribute to the Pathogenesis of Neurological Disorders

GABAergic astrocytes play a part in the pathogenesis of many diseases including Alzheimer’s disease (AD), stroke, epilepsy, and other neurological diseases.

In middle age AD mice, the size of astrocytes increases significantly. Astrocytic GABA in the cortex and dentate gyrus showed an approximately normal distribution with animals’ age. In normal mice, only a brief increase in GABA levels occurred in middle age. Excessive GABA is found accumulated in and released from astrocytes even in absence of amyloidosis in AD mice (Brawek et al., 2018). In the dentate gyrus of AD mice, astrocyte-derived GABA inhibits the number of ridges of granulosa cells, therefore impairing cognitive functions (Jo et al., 2014). The blockage of GABA production and release in reactive astrocytes restores the memory of AD mice (Jo et al., 2014). An abnormal amount of GABA released by astrocytes was also observed in AD patient samples (Le Meur et al., 2012). In addition, human hippocampal astrocytes release GABA to induce slow outward currents (SOCs) of neurons, leading to neurotransmission inhibition.

Besides neurodegenerative diseases, GABAergic astrocytes dysfunction is involved in other neurological disorders. In stroke, the internal capsular infarct induces reactive astrocyte proliferation and GABA release in the motor cortex (Nam et al., 2020). Reactive astrocyte-derived GABA inhibits neuronal glucose metabolism, which can be erased by inhibiting GABA synthase MAO-B. Therefore, MAO-B inhibitor combined with rehabilitation therapy may be a new strategy to promote functional recovery after stroke. In epilepsy, GABA has been found to be progressively accumulated in reactive astrocytes (Müller et al., 2020). The overproduction of GABA in reactive astrocytes is mediated by both decarboxylation of glutamate and putrescine degradation, and the excessive release of GABA preserves tonic inhibitory currents in the epileptic brain (Eid et al., 2013; Müller et al., 2020). In depression, the blockage of GABA synthesis in astrocytes restores prominent plasticity in the prefrontal cortex in depressed rats (Srivastava et al., 2020). Under acute hyperammonemia, the release of astrocyte GABA induced by α-2 adrenergic receptor agonists plays a neuroprotective role (Gaidin et al., 2020b). One possible mechanism is that astrocyte GABA act on GABAceptive microglia with GABA_A_Rs and GABA_B_Rs, which may inhibit microglial activation and alleviate neuroinflammation (Malaguarnera et al., 2021).

## Future Directions to Combine with Current Research Hotspots

Although mounting evidence has indicated important roles of astrocytes as GABAceptive and GABAergic cells in the regulation of neural functions, there are still many questions to be addressed. Here, we summarize current research hotspots and provide our thoughts that may inspire future studies.

### Are Exosomes Able to Mediate GABAceptive and GABAergic Astrocyte-Dependent Regulation of Neuronal Cells?

Exosomes, a subtype of small bilipid layer extracellular vesicles (EVs), serve as an essential regulator of neural functions (Chivet et al., 2012, 2014). We recently proposed a model that exosomes might serve as novel neurotransmitters (Xia et al., 2022). Given that, whether exosomes mediate GABAceptive and GABAergic astrocyte-dependent regulation of neuronal cells has emerged as an interesting question. It has been reported that exosomes derived from GABA-treated intestinal cells or from the serum of GABA-treated mice are able to activate neuronal cells *in vitro* by affecting the expression of genes related to memory in the hippocampus (Inotsuka et al., 2021). Besides, although in the absence of direct causal evidence, hyperactivation of GABA receptors and the abnormal release of exosomes have been closely linked to neurological disorders including epilepsy (Khalyfa and Sanz-Rubio, 2019). These studies implied a positive answer to this question, which needs to be exhaustively examined in future works.

### Is GABA Able to Regulate Inflammatory Responses of Astrocytes?

Since the first publication that proposed an A1/A2 model for reactive astrocytes in 2017, this field has explosively expanded (Liddelow et al., 2017). Currently, multiple neurotoxic or inflammatory stimuli have been identified to trigger A1 or A2-like reactive phenotypes of astrocytes (Li et al., 2019; Peng et al., 2020). However, the roles that GABA plays in astrocyte inflammatory responses remain controversial. It has been reported that GABA receptors participate in the activation of astrocytes post lipopolysaccharide (LPS) and interferon-gamma (IFNγ) stimulation (Lee et al., 2011). This study implicated GABA as an anti-inflammatory molecule that decreases astroglial activation and inhibits pro-inflammatory pathways. In contrast, another study has demonstrated that GABA treatment and the subsequent activation of GABA_A_Rs induce activation of astrocytes, ascertained by enhanced expression of GFAP (Runquist and Alonso, 2003). These conflicting observations indicate that astrocytes are a highly heterogeneous population and the effects of GABA on astrocyte inflammatory responses are highly dynamic. Therefore, more comprehensive investigations are urgently needed to expand our understanding in this field.

### Is GABA Able to Mediate Metabolic Reprogramming of Astrocytes?

Metabolic reprogramming is the alteration of energy metabolism modes that were firstly reported in cancer cells. Cancer cells can switch their metabolism mode to a glycolytic one even under aerobic conditions for rapid energy generation. This switch meets cancer cells’ bioenergetic and biosynthetic demands to support their rapid proliferation (Ward and Thompson, 2012). Hence, cancer cells get energy *via* high consumption of glucose and its conversion into lactic acid by glycolysis mostly, whereas normal cells mainly utilize mitochondrial oxidative phosphorylation (Biswas, 2015). Recent studies reveal that activated normal cells also undergo a distinct metabolic shift that significantly impacts their biological functions. Under resting conditions, astrocytes in adult brains almost exclusively utilize the complete oxidative metabolism of glucose for energy supply (Hertz, 2011). Under other conditions, however, astrocytes have the capacity to switch to a mode with a high glycolytic rate and lower oxidative metabolism as evidenced by the high expression of 6-phosphofructo-2-kinase/fructose-2, 6-biphosphatase 3 (Pfkfb3), a key positive modulator of glycolysis, and low activities of pyruvate dehydrogenase (PDH), the enzymatic complex that generates TCA cycle substrate acetyl-CoA (Herrero-Mendez et al., 2009; Halim et al., 2010). These observations reveal that the metabolic states of astrocytes are altered with environmental changes. Interestingly, 2-deoxy-D-glucose (2-DG), a glucose analog that inhibits glycolytic enzymes, has been reported to potentiate GABAergic tonic inhibition *via* neurosteroid-mediated activation of extrasynaptic GABA_A_Rs in the brain granule cells (Forte et al., 2016). A similar phenomenon may exist in astrocytes as well. In addition, the integration between glycolysis and the glutamate-glutamine cycle has been reported to participate in the regulation of astroglial activation (Hertz and Chen, 2017). Since GABA is the substrate and product of glutamate-glutamine cycle, intracellular GABA is likely to modulate glycolytic rates of astrocytes *via* modulating glutamate production (Cabrera-Pastor et al., 2019). Therefore, the potential reciprocal regulation between GABA and metabolic reprogramming may significantly manipulate astrocyte functions, especially under inflammatory conditions.

In summary, there are knowledge gaps in current understandings of the functions and regulations of GABAceptive and GABAergic astrocytes. More in-depth and systematic researches are needed to unmask the unknowns of GABAceptive and GABAergic astrocytes in the future.

## Conclusions

Recent studies identified GABA as a novel gliotransmitter in the CNS. The activities of GABAceptive astrocytes driven by inhibitory cells regulate both inhibitory and excitatory neurotransmission. More importantly, astrocytes themselves produce and release GABA to influence the brain function directly. The GABAceptive and GABAergic features make astrocytes a key regulator in both the maintenance of the proper function of the CNS and the pathogenesis of various neurological disorders. More comprehensive investigations will unveil the physiological and pathological roles of GABAceptive and GABAergic astrocytes yet to be discovered, and will greatly promote the progress of neuroscience to shed light on the development of novel astrocyte-dependent therapeutic strategies in treating neurological disorders.

## Author Contributions

JL, XX, and JZ conceived the manuscript. JL, XF, and XX collected references. JL, XF, XX, and YW wrote the manuscript. All authors contributed to the article and approved the submitted version.

## Conflict of Interest

The authors declare that the research was conducted in the absence of any commercial or financial relationships that could be construed as a potential conflict of interest.

## Publisher’s Note

All claims expressed in this article are solely those of the authors and do not necessarily represent those of their affiliated organizations, or those of the publisher, the editors and the reviewers. Any product that may be evaluated in this article, or claim that may be made by its manufacturer, is not guaranteed or endorsed by the publisher.

## Abbreviations

2-DG: 2-deoxy-D-glucose
7TM: Heptahelical transmembrane
AC: Adenylyl cyclase
ACBP: acyl-CoA-binding protein
AgRP: Agouti-related protein
Aldh1a1: Aldehyde dehydrogenase 1 family A1
ATP: Adenosine triphosphate
BBB: Blood-brain-barrier
BF: Basal forebrain
BGT-1: Betaine-GABA transporter
BZR: Benzodiazepine receptor
CAOs: Copper amine oxidases
CNS: Central nervous system
CRD: Cysteine rich domain
CTR1: Copper transporter
Cx43: Connexin 43
DA: Dopamine
DAO: Diamine oxidase
DBI: Diazepam-binding inhibitor
EAAT: Glutamate transporter
EVs: Extracellular vesicles
GABA: Gamma-aminobutyric acid
GABA_A_Rs: GABA_A_ receptors
GABA_B_Rs: GABA_B_ receptors
GAD: Glutamic acid decarboxylase
GAT: GABA transporter protein
GFAP: Glial fibrillary acidic protein
GPCR: G-protein coupled receptors
IFNγ: Interferon-gamma
KCC2: Potassium chloride cotransporter 2
LPS: Lipopolysaccharide
MAO-B: Monoamine oxidase B
mGluR: Metabotropic glutamate receptor
mPFC: Medial prefrontal cortex
nRT: Thalamic reticular nucleus
ODN: Octadecaneuropeptide
PDH: Pyruvate dehydrogenase
Pfkfb3: 6-phosphofructo-2-kinase/fructose-2, 6-biphosphatase 3
PGE2: Prostaglandin E_2_
PV: Parvalbumin
SOCs: Slow outward currents
SOM-INs: Somatostatin-expressing interneurons
TSPO: Translocator protein
TTN: Triakontatetraneuropeptide
VFT: Venus flytrap
VGCC: Voltage-gated calcium channels.
